# Adaptability and growth of *Hippocampus kuda* and *Oryzias melastigma* under rapid temperature changes

**DOI:** 10.3389/fphys.2024.1464123

**Published:** 2024-09-19

**Authors:** Yongjian Xu, Penghong Lin, Wenxin Zhang, Xia Pan, Jun Lu, Yang Bo

**Affiliations:** Key Laboratory of Aquacultural Biotechnology of MOE, School of Marine Science, Ningbo University, Ningbo, China

**Keywords:** *Hippocampus kuda*, *Oryzias melastigma*, rapid temperature change, adaptability and growth, precision aquaculture

## Abstract

Temperature changes had a huge impact on the growth of aquaculture organisms, which mainly involved two parameters: the changing amplitude and the changing speed. Wide-adaptability and narrow-adaptability were divided by the amplitude, while fast-adaptability and slow-adaptability proposed in this article were divided based on the speed. Investigating the impact of the changing speed on artificial farming was vital. In this study, two fish species of wide-adaptability, *Hippocampus kuda* and *Oryzias melatigma*, were selected as research objects, explored the effects of temperature changing speeds on them under 2 changing amplitudes of 2°C and 4°C. The similarities and differences in their responses to temperature changes were analyzed and compared from the aspects of feeding, metabolism, physiology, immunity, and growth. The results showed that all 3 changing speeds (0.5°C/h, 1°C/h, and direct input) had no effect on the growth of *O*. *melatigma* under the 2°C amplitude, while there were significant differences in various aspects of *H. kuda* in the treatments with the speeds between 0.5°C/h and direct input, such as a significant difference in growth, in food intake, and in response speeds and response levels of several enzymes and related genes. Under 4°C amplitude, the impact of all 4 changing speeds (0.5°C/h, 1°C/h, 2°C/h and direct input) on both fish was more pronounced. *H. kuda* showed a significant difference of growth among 3 groups, and the critical safe speed was about 0.5°C/h in its heating treatments. And the growth decrease only occured the heating treatment of direct input in *O*. *melatigma*. Furthermore, some genes responded quickly and efficiently to the low-speed changes of temperature in *H. kuda*, but were inhibited in the treatments with high-speed changes. However, they can still express rapidly and efficiently in the high-speed treatments of *O*. *melatigma*, included several stress-related genes, lipid metabolic-related genes, and immune-related genes. Seen from these differences, the energy source used in *H. kuda* to resist stress was single and short-lived. So, under a long-term stress, *H. kuda* gradually transformed from normal physiological stress into pathological stress, leading to the outbreak of diseases. Therefore, for precise aquaculture of *H. kuda*, stricter and more precise control of environmental temperature is necessary to prevent rapid and big temperature changes from affecting the growth and survival of the seahorse.

## 1 Introduction

Throughout the whole life history of fish, water temperature is one of the crucial non-biological factors for its growth and development. Changes in water temperature can affect its physiological processes such as feeding, digestion, metabolism, and reproduction. Generally, temperature affects organisms in all aspects (Alfonso et al., 2021, p.1496), included oxidative stress, e.g., changes in activity or content of superoxide dismutase (SOD), Catalase (CAT), malondialdehyde (MDA) and peroxidase (POX) (Kato, 1997, p.25), digestibility and metabolism, e.g., α-Amylase (AMS), lipase (LPS) and proteinase (Hu et al., 2016, p.13; Pan et al., 2022, p.1084), non-specific immunity, e.g., alkaline phosphatase (AKP) and acid phosphatase (ACP) (Xiang and Liu, 2007, p.396; Dittmar et al., 2014, p.744; Makrinos and Bowden, 2016, p.50; Wang et al., 2021, p.e73776), and growth and development (Ye et al., 2016, p.101; Burggren et al., 2019, p.732; Lu et al., 2020, p.e73547), and so on. Therefore, investigating the effects of temperature changes is of great significance for fish farming and provides important guarantees for the success of aquaculture (Xu, 2019, p.1; Lu et al., 2023, p.e739114).

Previously, the fish was divided into widely-adapted and narrowly-adapted types based on its ability to adapt to temperature changes. This classification had a good guiding role in selecting suitable breeding species. However, it only investigated the amplitude of adaptation to temperature changes, and does not fully reflect the impact of the changing speed of temperature. Following the above division, we divided the fish into slowly-adapted and fast-adapted types based on its ability to adapt to temperature changing speeds. The slow-adaption referred to being sensitive to temperature fluctuations, and its growth and physiology was easily affected by rapid temperature changes. The fast-adaption was less sensitive. For example, *Hippocampus kuda* was a widely-adapted fish with an amplitude of 6°C–32°C (Mu et al., 2017, p.355). And, *Oryzias melatigma*, known as Indian madaka, also was a widely-adapted fish (4°C–35°C, Murata, et al., 2020, p.59). However, there was a significant difference in their performance in adapting to rapid temperature changes. Previous researches in our laboratory had found that within the temperature range of 22°C–28°C, there was a 70% probability of anorexia reaction of *H*. *kuda* occurred under the temperature change of 2°C within 2 h, and part individuals even developed into enteritis and died. While *O*. *melatigma* had no effect at the speed of 4°C/h. It can be preliminarily determined that *H*. *kuda* was a slowly-adapted fish, and *O*. *melatigma* was a fast-adapted fish. This phenomenon was also reflected in their artificial farming, where the seahorse was characterized by strict environmental requirements and difficult to successful farming (Lv et al., 2003, p.46; Wang et al., 2020, p.730; Xie et al., 2020, p.e735168). And *O*. *melatigma* was so easy to be farmed. There were also many reports of other organisms being affected by sudden changes of temperature. included the non-specific immunity of sea cucumber (Liu et al., 2013a, p.1342), the survival of *Panulirus homarus* (Huang et al., 2017, p.5973), the food conversion rate of sea urchins (Siikavuopio et al., 2012, p.27), the antioxidant capacity and immunity of marine medaka (Wang et al., 2019, p.378), and the feeding behavior of California moray prey (Moretto, et al., 2022, p.126030), and so on.

Therefore, it is necessary and important to pay attention to the adaptability of farmed organisms to rapid changes in environmental factors, and finds safety measures to avoid risks, in order to improve aquaculture technology. This study explored the responses of *H. kuda* and *O*. *melatigma* to rapid temperature changes, compared their similarities and differences in growth, behavior, physiological and biochemical aspects, and expressions of several functional genes, then analyzed the response mechanisms and self-regulation characteristics of fast-adapted and slowly-adapted, and promoted aquaculture technology of fish farming.

## 2 Materials and methods

### 2.1 Experimental fish

The seahorse of *H. kuda* (body length 4.96 ± 0.34 cm, weight 0.276 ± 0.031 g) was provided by Xiangshan Base of Ningbo Yonghe Biotechnology Company, and temporarily raised for 2 weeks at the pilot base of Meishan campus, Ningbo University. Individuals with clean body and good vitality were randomly selected for the experiment. *O*. *melatigma* (body length 3.12 ± 0.46 cm, weight 1.730 ± 0.243 g) was bred in our laboratory in Meishan campus, also randomly selected for the experiment. Both fishes had similar ages of about 45 days old.

The temporary breeding conditions were: water temperature of 25°C ± 0.5°C, salinity of 25, dissolved oxygen (DO) >5 mg/L, light intensity of 1000lx, and photoperiod of 14L:10D. During the periods of temporary breeding and experiment, *H. kuda* was fed frozen *Mysis* (Tianjin Fengnian Aquaculture Company, China) with a total length of 1–1.5 cm as bait, and *O*. *melatigma* was fed with artificial formula feed (Ningbo Tianbang Group, China). Fed once a day at 8:00 Am, and removed any remained bait and feces after 2 h of feeding. The seawater was exchanged approximately 50% before feeding.

### 2.2 Experimental design

The experiment consisted of two tests.

Test 1: The set temperature changing amplitude was ±2°C. There were 3 groups of breeding temperature in the test, included the control group (Ck) with the temperature of 25°C, the heating group (HG) with an increase of 2°C (27°C) and the cooling group (CG) with a decrease of 2°C (23°C). There were 3 ways to reach the set temperatures (from 25°C to 23 or 27°C), that was 3 types of changing speeds: direct input (Marked as 0H2±), increasing or decreasing 2°C within 2 h (2H2±), where the changing speed was 1°C/h, and changing 2°C within 4 h (4H2±, 0.5°C/h).

Test 2: The set temperature changing amplitude was ±4°C. There were 3 groups of breeding temperature (25°C, 29°C, 21°C) and 4 speed treatments of 0H4± (direct input), 2 h (2H4±, 2°C/h), 4 h (4H4±, 1°C/h), and 8 h (8H4±, 0.5°C/h).

The temperature changing process was carried out in controlled-temperature illuminated incubators (Jiangnan GXM-508A, Ningbo, China). By conducting pilot experiments, appropriate temperature changed conditions were identified to ensure that the temperature changing amplitude was achieved within the required time (not sure at a uniform rate). The air temperature inside the incubator was controlled within the variable range of ±0.5°C, and the water temperature fluctuation was less than ±0.2°C in the incubator. After the temperature changes completed, it would be maintained the target temperature for 7 days before ending the experiment. In the experiment, the salinity of each treatment remained at 25, with three replicates for each treatment.

### 2.3 Experimental methods

#### 2.3.1 Analysis of related gene expression

The tissue samples were taken from *H. kuda* and *O*. *melatigma* at 12 h, 4 day, and 7 day after the temperature changed completeness in each treatment. During the sampling process, the fish were anesthetized, and their liver tissues from 2 individuals were taken from each replicate (a total of 6 individuals), then merged as one sample. Total RNA was extracted from each sample using the Trizol method, followed by real-time fluorescence quantitative PCR analysis (Pan et al., 2020, p.1421; Pan et al., 2022, p.1084).

According to literature reports and our previous researches, 16 genes were selected in *H. kuda*, and another 16 genes were selected in *O*. *melatigma* as candidate genes for the response analysis. Their specific primers were designed with the software of Primer Premier 5, using mRNA sequences from NCBI. Alternatively, based on the reported Unigene sequences in the transcriptome library, the primer sequences of all genes were shown in Table 1. At the same time, *b2m* and *actb* were used as an internal reference gene for gene expression analysis in *H. kuda* and *O*. *melatigma*, respectively. The relative expression levels of each target genes were calculated using Livak analysis method, and the 2^−ΔΔCT^ method was used to calculate the relative expression of the target gene.

**TABLE 1:** The names and primer sequences of 16 genes analyzed in *H. kuda* and *O*. *melastigma*

	*Hippocampus kuda*	*Oryzias melastigma*
Gene name	Primer sequences (5′-3′)	Primer sequences (5′-3′)
Stress-related genes		
*Sod*	F: TCACATACTTCACGGGTTTCG R: AGGGAAATGTTCAAGGTACTGC	F:CAAATGGGTGTACCAGTGCG R:ATCTCATCATCTCCTGCGGTC
*Gst*	F: AAATGACTCTGTACTGGGGCG R: CCCCTGGGGTTAATATCGAGC	F: GGAAGACAGCTACGGACAGC R: GAGTTCCTCCTGGTCTTGCA
*Hsp70*	F: GTCGGTGAAATAACAGGGAACA R: CTCTGGGTCTACAGGTATTAAGGTG	F: ATCAGGAGACACCCACCTCG R: GCCCTCTTGTTCTGGCTAATGT
*Hsp90*	F: GGACTTCTACTCGGGCTGCTA R: TCCTCCTCCCTCTGAACCAC	F: CGGGCTTCTTTGTGCACTTC R: CTGCATTCAAGCCATCGAGC
Metabolic-related genes		
*Idh3b*	F: TTGCCCGTCATCTTCATCTG R: GACATCCATACCATCCACCCT	F:TCGCTGTGGAAAGGCTCTAA R:CTCTTCTGGGCTGAAGTAAACG
*Mdh1*	F: TCTGCGACCACATGAGGGA R: TCTGGACGGGGAAGGAGTAG	F:GCCGCTCAGAAGCTATTTGC R:GCCTCCTGTCCTCTAGTAGCA
*Cpt1*	F: TCAGGGCCAGACGATGCTT R: CGACCGTGCTGCTCAAACA	F:ACCGACGCCATTCCCATA R:CTGTCCAGAACCTCCACATACC
*Fas*	F: GTCCCATTGTGCTGTTGTGAC R: CGGACTCCTGAATATCCAGCC	F:TGCCACTCCTCCATCTTTGA R:ACGGTTGCTGTAGCCGAAC
Growth-related genes		
*Mstn*	F: AATAATCCAGTCCCAGCCGAAG R: CCCGACCTCACCAAGCACAACA	F: CCACGCTTTGACAGCGAAAA R: CTGCGCGTGCATAAATGAGC
*Gh*	F: ATTATCAAAGTCTGGGAGGC R: GGTAGGTCTCCACCTTGTGC	F: TCCAGAACCTTTCAGACGCC R: CCACATGGTCGTGTGCTCTT
Immune-related genes		
*Gadd45α*	F: CTTTGGAAGGGACGTAGGCA R: AAACATCCGCAGAGGAGTGAA	F:ATGTAGCTGACCCGAGGACT R:GTGACTCGGTTCTGTTCAGACT
*Il-10*	F: GGAGGACACGAGGGACTTGA R: GCCTTTGTTTTGCATCTGACTG	F: GAGTTCGCTTGCCAGACCAT R: GACAGCACATGCGGCTTTAC
*Bcl-2*	F: GTGAGGTACGTGCCGATGGT R: GGGCTGGGATGCTTTTGTG	F: ACGTGTCTCATCGCAGTGTT R: ACCGAGCGAGCCGTTATTAG
*P53*	F: CTTCATCTCATTTCCCAGCATCT R: GGCTTCTAAACCCCACCCTCT	F:GCCTTGAAAAGTCTCCATCTGC R:TTCTTCCTCCGTTTTGCGGT
*Casp9*	F: GGTTCTCCGAATCAGATGGTG R: TGCGGTTCAGGGGTCAAT	F:CCAAGACCCATCAAGGTCCA R:TTCAGTTCGCTCTTCGGCTC
*Casp3*	F: GGGACGGATGTTGATGCTG R: TGGTCCTCTTGGGATACACTCA	F: TCTTGTGGGGAAACCAAAGC R: ATAGCCTGAAACGGTGGAGTAG
*b2m/actb*	F: TACACCCACCAGCCAGGAAA R: GGACTCGACGACATCGAACATC	F:CCCAAAGCCAACAGGGAGA R:CAGAGGCATACAGGGACAGCA

#### 2.3.2 Analysis of related enzyme activity

The visceral tissue (without liver) was used for enzyme activity analysis. Accurately weighed 1 g tissue, added 9 ML 0.1 mol/L pH 7.4 phosphate buffer, mechanically homogenized (DIRAD DR-310, Jiangsu, China) under ice bath, centrifuged at 2,500 rpm for 10 min in a high-speed centrifuge (Eppendorf 5430R, Germany), and extracted the supernatant. The supermatant would be diluted with the phosphate buffer, and for determination of the following enzymes. Superoxide dismutase (SOD) by the Hydroxylamine method, malondialdehyde (MDA) by the Thiobarbituric acid (TBA) method, α-Amylase (AMS) by the Starch-iodine colorimetric method, lipase (LPS) by the Methyl resorufin substrate method, alkaline phosphatase (AKP) and acid phosphatase (ACP) by the Disodium phenyl phosphate method. The above analysis was carried out using the corresponding reagent kits from Nanjing Jiancheng Biotechnology Institute (Nanjing, China) according to the instructions. The protein content was measure with Coomassie brilliant blue staining (Candiano et al., 2004, p.1327; Yuan et al., 2024, p.1403391).

#### 2.3.3 Analysis of the RNA/DNA ratio

According to Buckly’s (1979) analysis method, the RNA/DNA ratio was anaylzed for showing the growth of both fish. Take respectively an appropriate amount of muscle tissue from the 4-day and 7-day samples and mix it with Tris buffer (0.05 mol/L, pH = 7.4) for homogenization. Suction 1.4 mL of the homogenization solution and mix it with 0.7 mL of 0.6 mol/L HClO_4_. Cool it on ice bath for 15 min, centrifuge at 4°C (12,000 r/min) for 10 min, and then remove the supernatant. Wash the precipitate in 1.12 mL of 0.3 mol/L KOH at 37°C in water bath for 1 h and then in ice bath for 30 min, and centrifuge again. Take out the supernatant and measure its absorbance value at 260 nm (Mettler UV5 nano, Germany), which was the absorbance value of RNA. At the same time, the precipitate was washed with 2.0 mL 0.2 mol/L HClO_4_, after centrifugation, the supernatant was removed. Then add 2.2 mL of 0.6 mol/L HClO_4_ to the precipitate and incubate at 85°C for 15 min, followed by an ice bath for 15 min. Centrifuge again, the precipitate was protein. Suction the supernatant and measure its absorbance value at 260 nm, which was the absorbance value of DNA. Thus, the ratio of RNA/DNA can be calculated.

#### 2.3.4 Analysis of feeding behavior

On the 4th and 7th days, 6-8 individuals of fish were randomly selected from each treatment for observing their feeding behavior. A glass aquarium (L15 cm × W10 cm × H13 cm) was used for behavioral photography. Two Panasonic VX1 portable cameras (Panasonic Co., Japan) were placed directly above and in front of the aquarium for recording the feeding behavior, in order to subsequent repeated observation and analysis. The water temperature, salinity, light intensity and other conditions inside the aquarium were the same as the original experimental environment. The specific steps in the entire feeding process were as follows: first, let an individual adapt to the new environment in the aquarium for 60 min, and then fed live *Artemia* larvae with a density of 1 ind./mL. The cameras started recording 5 min before feeding and lasted for 45 min. The following 3 parameters of feeding behavior were analyzed in each treatment based on the recorded videos.

Feeding response time (second): the period time elapsed from the entry of *Artemia* into the water to the first larvae was swallowed by fish.

Feeding rate (ind./min): The average rate of *Artemia* consumed by fish during a 10-min period from the 11th minute to the 20th minute.

Food intake (individual): The total number of *Artemia* consumed by fish throughout the feeding.

### 2.4 Data processing and statistical analysis

Statistical analysis of experimental data was conducted using SPSS 22.0 (IBM Co., NY, United States). Two-way ANOVA was used to examine the effects of temperature changes in amplitude and in speed on the growth, feeding behavior, gene expression levels, and enzyme activity of both fish. T-test was used to analyze the effects of different temperature changes and compare the differences in average values within and between 2 species fish. The data were expressed as mean ± standard error, with P < 0.05 indicating significant differences. Use GraphPad Prism 8.0 to plot data and statistical results.

## 3 Results

### 3.1 Effects on RNA/DNA ratio

The variations of RNA/DNA ratios in their muscles of *H. kuda* and *O*. *melastigma* under different changing amplitudes and speeds of temperature were shown in Table 2.

**TABLE 2:** Variations of RNA/DNA ratio in the muscle of *H*. *kuda* and *O*. *melastigma* in all treatments.

Treatment (°C)	Sampling day	CK(25°C)	4H2	2H2	0H2
kuda −2	4d	2.221 ± 0.216^aA^	2.212 ± 0.207^aA^	1.962 ± 0.221^abA^	1.860 ± 0.170^bB^
	7d	2.362 ± 0.295^aA^	2.395 ± 0.243^aA^	2.425 ± 0.237^aA^	2.304 ± 0.178^aA^
kuda +2	4d		1.995 ± 0.185^abA^	1.931 ± 0.203^abA^	1.747 ± 0.295^bcA^
	7d		2.306 ± 0.252^aA^	2.194 ± 0.167^aA^	1.584 ± 0.226^cA^
madaka-2	4d	2.626 ± 0.213^aA^	2.561 ± 0.291^aA^	2.474 ± 0.126^abA^	2.395 ± 0.174^abA^
	7d	2.665 ± 0.347^aA^	2.664 ± 0.145^aA^	2.621 ± 0.133^aA^	2.471 ± 0.206^abA^
madaka+2	4d		2.343 ± 0.288^abA^	2.329 ± 0.176^abA^	2.200 ± 0.325^bA^
	7d		2.618 ± 0.321^aA^	2.403 ± 0.219^abA^	2.455 ± 0.175^abA^

	Sampling day	8H4	4H4	2H4	0H4
kuda −4	4d	1.963 ± 0.187^abA^	1.738 ± 0.273^bcA^	1.602 ± 0.164^bcA^	1.325 ± 0.117^cdA^
	7d	2.238 ± 0.332^abA^	1.912 ± 0.187^abA^	1.582 ± 0.153^bcA^	1.468 ± 0.136^cA^
kuda +4	4d	1.823 ± 0.163^bB^	1.657 ± 0.183^bcA^	1.466 ± 0.158^cdA^	1.157 ± 0.245^dB^
	7d	1.857 ± 0.226^bB^	1.734 ± 0.186^bcA^	1.334 ± 0.174^cdA^	1.238 ± 0.226^dB^
madaka-4	4d	2.671 ± 0.286^aA^	2.665 ± 0.259^aA^	2.449 ± 0.262^aA^	2.032 ± 0.187^bcA^
	7d	2.632 ± 0.313^aA^	2.707 ± 0.263^aA^	2.587 ± 0.251^aA^	2.474 ± 0.278^aB^
madaka+4	4d	2.635 ± 0.247^aA^	2.417 ± 0.202^abA^	1.977 ± 0.167^bcB^	1.736 ± 0.243^cA^
	7d	2.614 ± 0.228^aA^	2.369 ± 0.276^abA^	2.353 ± 0.241b^aA^	1.817 ± 0.195^bcA^

Note: Different lowercase letters indicate significant differences among different treatments with the same fish at the same week (P < 0.05); Different capital letters indicate significant differences in the same treatment at different week (*P* < 0.05).

Under 2°C amplitude, there was a mild difference on the RNA/DNA ratios among 3 groups with different speeds in *H. kuda*, and no difference on *O*. *melastigma* at all, compared with their CK, respectively. The ratios of *H. kuda* in 3 treatments of 0H2± and 2H2+ had significantly decreased on the 4th day (P < 0.05). And 0H2- and 2H2+ had recovered on the 7th day, but 0H2+ became worse (Table 2). Under 4°C amplitude, there was a significant effect on the RNA/DNA ratios among 3 groups with different speeds in both *H. kuda* and *O*. *melastigma*. For *O*. *melastigma*, the 0H4- treatment on the 4th day was affected by a −4°C cooling (*P* < 0.05), and recovered on the 7th day. 2H4+ and 0H4+ were also affected by the heating (P < 0.05), and 2H4+ had recovered while 0H4+ was still suffered on the 7th day. For *H. kuda*, all treatments showed a significant decrease on the 4th day (*P* < 0.05). And 4H4± and 8H4± had recovered, while 2H4± and 0H4± became even worse on the 7th day. Comparing both fish, the impact of temperature changes on the growth of *H. kuda* was greater than that on *O*. *melastigma*. At the same time the impact of the same speed was different under different amplitudes, reflecting the synergistic effect of two factors on growth. Roughly summarized, the critical safe speed of *O*. *melastigma* was quite big, while that of *H. kuda* was around 1°C/h with 2°C amplitude.

### 3.2 Effects on feeding behavior

Three parameters of feeding behavior, included feeding response time, feeding rate, and food intake, were measured for both *H. kuda* and *O*. *melastigma* in each treatment on the 4th and 7th day. The variations of food intake were shown in Table 3.

**TABLE 3:** Comparisons of food consumption between *H*. *kuda* and *O*. *melastigma* in each treatments.

Treatment (°C)	Sampling day	CK(25°C)	4H2	2H2	0H2
kuda −2	4d	146.8 ± 15.36^aA^	138.2 ± 12.17^aA^	122.5 ± 21.34^abA^	91.2 ± 19.74^bA^
	7d	155.8 ± 14.68^aA^	145.8 ± 22.34^aA^	146.3 ± 27.66^aA^	125.3 ± 22.68^abA^
kuda +2	4d		110.6 ± 32.17^bB^	100.4 ± 22.34^bB^	61.3 ± 12.67^cA^
	7d		118.5 ± 23.37^abB^	122.7 ± 13.26^abB^	95.8 ± 31.62^bB^
madaka-2	4d	89.2 ± 15.38^aA^	101.2 ± 15.21^aA^	93.2 ± 15.67^aA^	86.3 ± 12.52^abA^
	7d	93.4 ± 11.33^aA^	89.3 ± 8.91^aA^	92.6 ± 17.68^aA^	82.4 ± 10.71^abA^
madaka+2	4d		98.3 ± 9.33^aA^	89.6 ± 11.27^aA^	76.9 ± 17.83^abA^
	7d		96.6 ± 11.87^aA^	82.7 ± 10.32^abA^	85.4 ± 18.94^abA^

	Sampling day	8H4	4H4	2H4	0H4
kuda −4	4d	97.3 ± 17.67^bA^	82.2 ± 16.34^bA^	37.8 ± 21.65^dA^	12.6 ± 7.44^eA^
	7d	138.4 ± 27.4^aB^	101.7 ± 19.53^bA^	63.4 ± 19.36^cB^	27.1 ± 11.27^dB^
kuda +4	4d	103.5 ± 34.33^bA^	67.7 ± 20.22^cA^	36.6 ± 24.85^dA^	4.7 ± 5.82^eA^
	7d	94.2 ± 35.62^bA^	40.3 ± 32.74^cdA^	23.4 ± 25.71^dA^	6.5 ± 6.33^eA^
madaka-4	4d	83.6 ± 15.42^abA^	97.5 ± 18.36^aA^	89.1 ± 15.62^aA^	65.8 ± 23.38^bB^
	7d	92.4 ± 12.53^aA^	86.7 ± 16.77^abA^	94.3 ± 13.71^aA^	91.1 ± 18.41^aA^
madaka+4	4d	80.9 ± 17.63^abA^	97.3 ± 22.25^aA^	74.7 ± 18.32^bB^	38.3 ± 24.21^cC^
	7d	86.3 ± 20.17^abA^	83.8 ± 21.71^abA^	95.2 ± 25.44^aA^	66.2 ± 22.34^bD^

Note: Different lowercase letters indicate significant differences among different treatments with the same fish at the same week (P < 0.05); Different capital letters indicate significant differences in the same treatment at different week (*P* < 0.05).

Overall, the variations in food intake of both fish were basically consistent with their growth. Under 2°C amplitude, different speeds of temperature changes had almost no effect on *O*. *melastigma* (*P*> 0.05), whether in heating or in cooling groups. For *H. kuda*, the food intake decreased obviously in 2 treatments of 0H2± on the 4th day (*P* < 0.05), and only 0H2- had recovered on the 7th day (Table 3). Under 4°C amplitude, the decreases in food intake of *O*. *melastigma* only occurred in 0H4± and 2H4+ treatments (*P* < 0.05). And all treatments (except for 0H4+) had recovered on the 7th day. However, the food intake of all *H. kuda* treatments had significantly decreased on the 4th day (*P* < 0.05). At the end of the experiment, only 8H4± had recovered, 2H4- had improved slightly, other 3 treatments of 0H4± and 2H4+ were even worse (Table 3). Comparing the treatments of the same speed under different amplitudes, it was evident that larger amplitudes had a greater impact on *H. kuda*.

By analysis, the other 2 feeding parameters also showed a similar characteritic, with the feeding response time prolonging and the feeding rate decreasing in *H. kuda*.

### 3.3 Effects on enzyme activity

**FIGURE 1:**
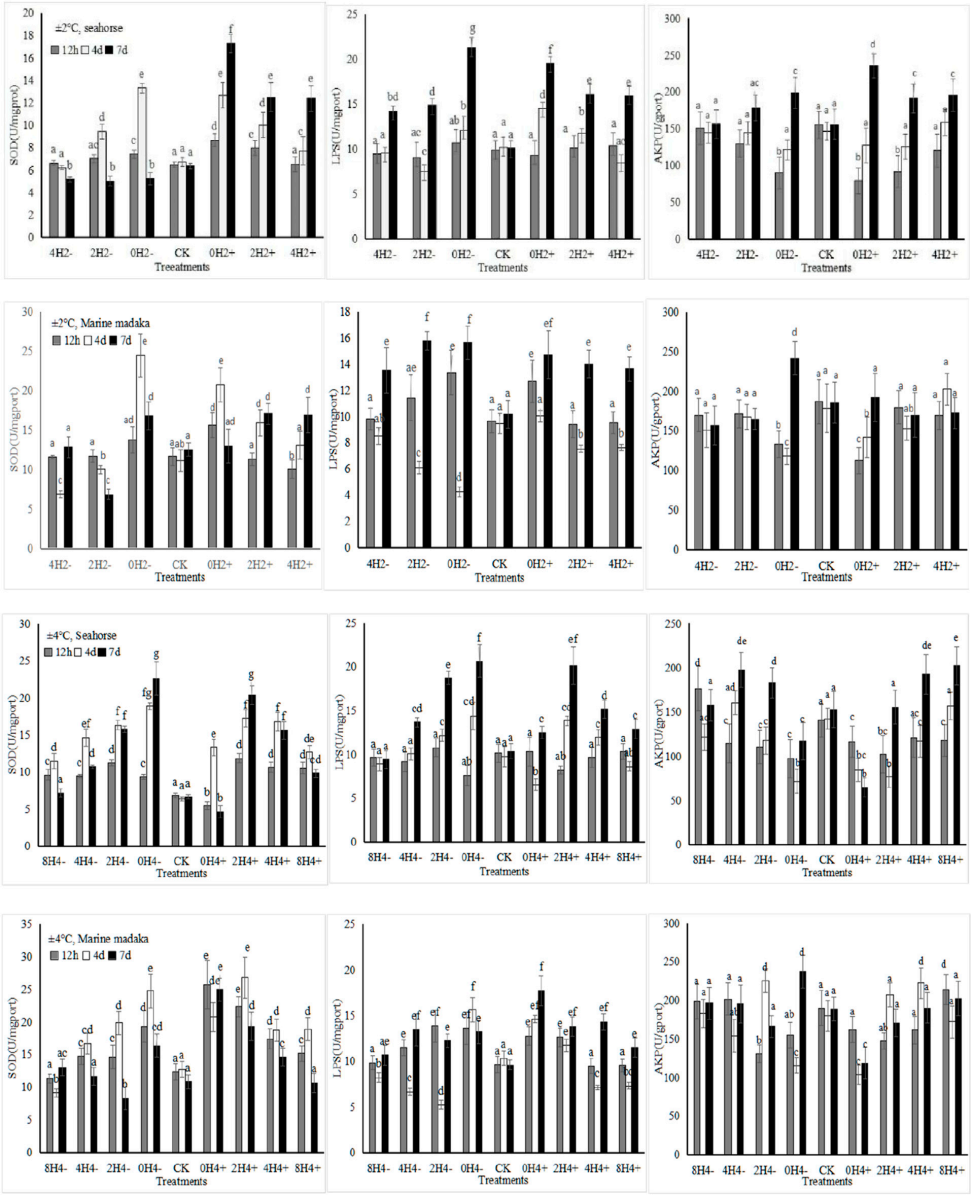
The effects of rapid temperature changes on SOD, LPS, and AKP activities in *H. kuda* and *O*. *melastigma*.

The activities or content of six enzymes (SOD, MDA, LPS, AMS, ACP, AKP) were analyzed in the experiment. The variations of SOD, LPS, and AKP in *H. kuda* and *O*. *melastigma* were shown in Figure 1.

Under both amplitudes, various enzymes showed their significant fluctuations in response to the different speeds of temperature changes, and their response trends were basically similar in *H. kuda* and *O*. *melastigma* (Figure 1). The bigger the amplitude, the greater the enzymes’ response was. And the faster the speed, the greater the enzymes’ response also was. At the same time, all enzymes also showed similar responses to temperature increasing/heating or deseasing/cooling, the impact of increasing temperature was greater. Certainly, there was some difference between *H. kuda* and *O*. *melastigma* at different speed with same amplitude, the main performance was that each enzyme had a difference in response time and response magnitude (Figure 1). Under 2°C amplitude, There was not much difference between 2 fish, except in 2H2- and 4H2+, where the enzyme expressions were slightly differed between 2H2- and 4H2+ in *H. kuda* (*P* < 0.05)*,* which did not occur in *O*. *melastigma*. Under 4°C amplitude, the difference was also reflected in the rapid response at 12 h, as well as the increasing number of treatments involved in the continuous increase in enzyme activities in *H. kuda* (Figure 1).

### 3.4 Effects on the expression of related genes

#### 3.4.1 Stress-related genes

**FIGURE 2:**
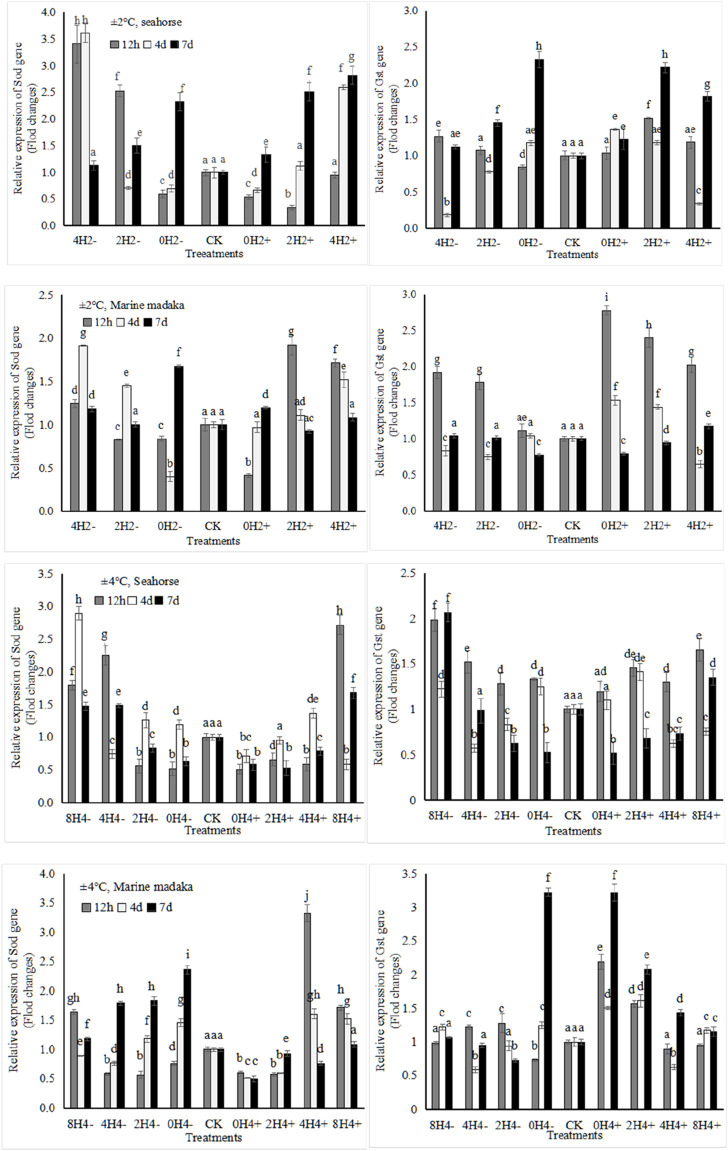
Variations in relative expression levels of *Sod* and *Gst* genes in *H. kuda* and *O*. *melastigma*.

The relative expression levels of 4 stress-related genes (*Sod, Gst, Hsp70, Hsp90*) were analyzed in *H. kuda* and *O*. *melastigma*. Their variations of *Sod* and *Gst* genes were shown in Figure 2.

Their variation trends in both fish were similar. They exhibited up and down oscillations at 12 h, 4 day, and 7 day after temperature changes, and gradually recovered in each treatment. The specific differences were shown as follows, under 2°C amplitude, *Sod* had a rapid expression of upregulation at 12 h in *O*. *melastigma*, but it had a slow response in *H. kuda*, even being inhibited (e.g., downregulation in 0H2± and 2H2+) or no response (4H2+) at 12h, and then upregulated at 4th and 7th days. *Gst* also exhibited a similar slow response in *H. kuda*. There was a greater difference between the 2 fish under 4°C amplitude. *Sod* showed its obvious different expression in the correspondent treatments between *H. kuda* and *O*. *melastigma* (*P* < 0.05), except in 0H4+, which was inhibited throughout the experiment. *Gst* was interesting, its response was similar to the control in all treatments of *O*. *melastigma* at 12h, except in 0H4± and 2H4+, where its expression was significant upregulated. *Gst* at 12 h also showed a rapid upregulation in all treatments of *H. kuda*, followed by up and down fluctuation in 8H4± and 4H4±, and continued to decrease in 0H4± and 2H4± on 4th and 7th day (Figure 2).

#### 3.4.2 Metabolic-related genes

**FIGURE 3:**
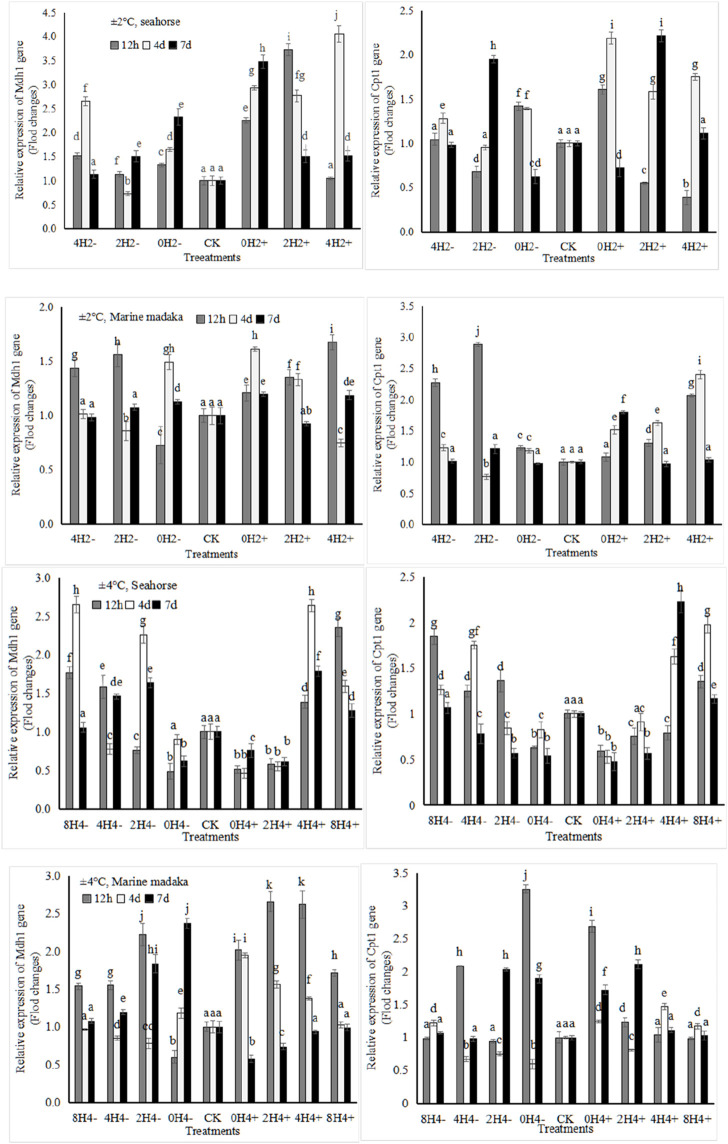
Variations in relative expression levels of *Mdh1* and *Cpt1* genes in *H. kuda* and *O*. *melastigma*.

The relative expression levels of 4 metabolic-related genes (*Mdh1, Idh3b, Cpt1, Fas*) were also analyzed in both fish. The expressions of *Mdh1,* involved in sugar-metabolism and *Cpt1,* involved in lipid-metabolism were shown in Figure 3.

Their variation trends were also similar in both fish. The specific differences were shown as follows, under 2°C amplitude, the *Mdh1* expression in *H. kuda* was generally higher than that in *O*. *melastigma*, especially in 0H2±, whose expression was significantly higher at 12 h (*P* < 0.05), and continued to rise on the 4 and 7 day. And it showed up and down fluctuations in 0H2± of *O*. *melastigma*. *Cpt1* showed a rapid upregulated response at 12 h in 0H2± of *H. kuda*, and had a similar performance in 4H2± and 2H2- of *O*. *melastigma* (Figure 3). Under 4°C amplitude, the *Mdh1* expression was nevertheless slightly upregulated in almost treatments of *O*. *melastigma*, especially had a significant upregulation at 12 h. In *H. kuda*, the upregulated expression of *Cpt1* only appeared in the slow-speed treatments (8H4± and 4H4±), while its downregulated response appeared in the high-speed treatments (0H4± and 2H4+). It can be seen that the expressions of both genes was similar in *H. kuda*, while their up-regulations only showed in the high-speed treatments of *O*. *melastigma*.

#### 3.4.3 Immune-related genes

**FIGURE 4:**
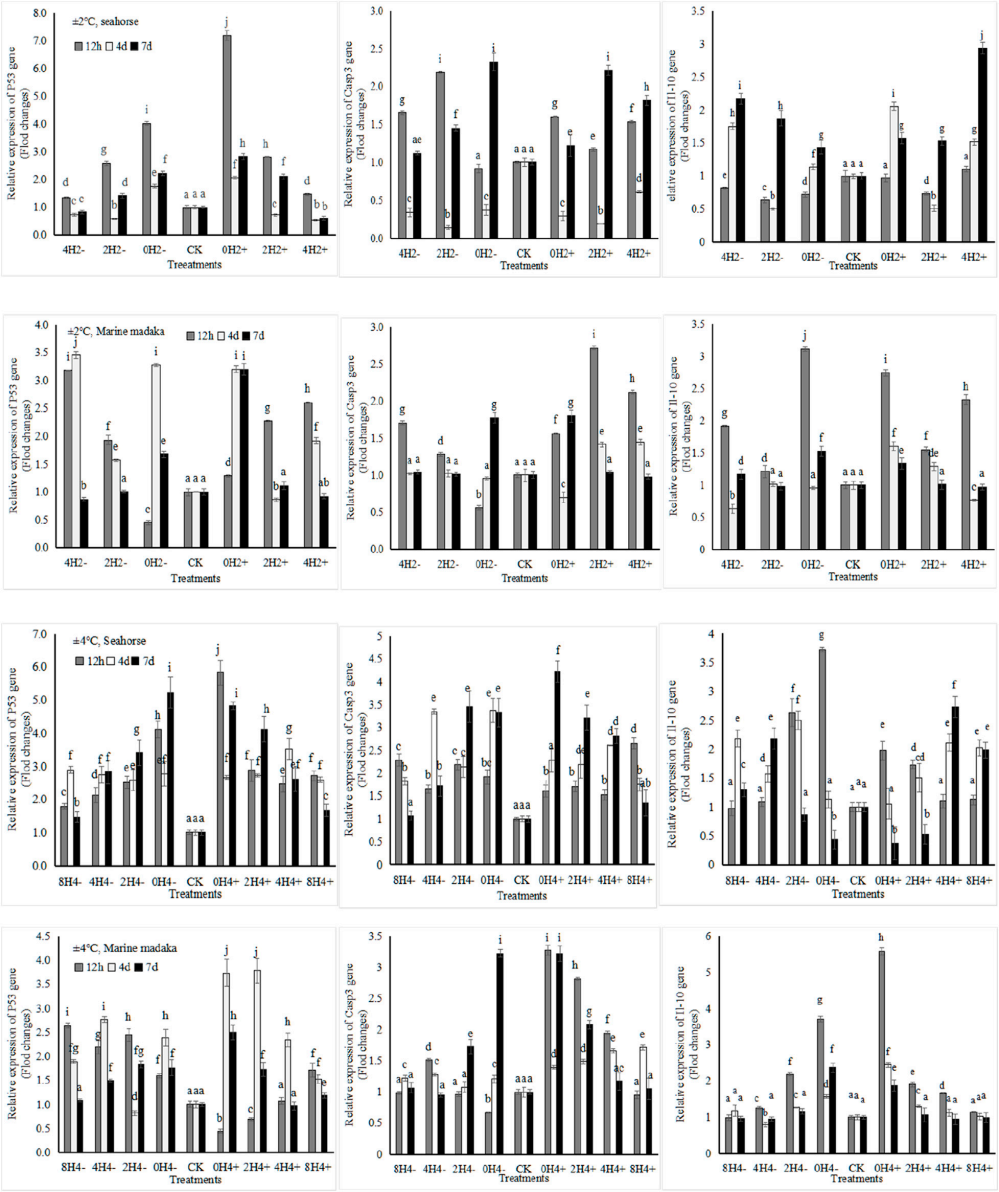
Variations in relative expression levels of *P53, Il-10* and *Casp3* in *H. kuda* and *O*. *melastigma*.

Six immune-related genes (*Bcl-2, P53, Gadd45α, Il-10, Casp3, and Casp9*) were analyzed in the experiment. And the variations of *P53, Il-10*, and *Casp3* in both fish were shown in Figure 4.

Their expressions were also similar between 2 fish, all showed up and down fluctuation, and gradually returned to normal with the extension of experiment in almost treatments.

The specific differences were, under 2°C amplitude, the expression level of *P53* at 12 h increased with the increasing speed in *H. kuda*, while it was the opposite in *O*. *melastigma*. *Casp3* was similar at 12 h between 2 fish. *Il-10* had a rapid upregulation at 12 h in *O*. *melastigma*, but no response in *H. kuda*. At the end of the experiment, All genes had nearly recovered in *O*. *melastigma*, except in 0H2±*.* But *Casp3* and *Il-10* had still at a high expression in *H. kuda*. Under 4°C amplitude, all genes had a rapid upregulation at 12 h in *H. kuda*, and kept still at a high expression levels on the 7th day, except for *Il-10* in 2H4± and 0H4±. In *O*. *melastigma*, *P53* had also a rapid response at 12 h in almost treatments, other 2 genes only happened in 2H4± and 0H ± 4. All genes had recovered in slow-speed treatments on the 7th day, but still needed time to recover in the high-speed treatments.

#### 3.4.4 Growth-related gene expression

**FIGURE 5:**
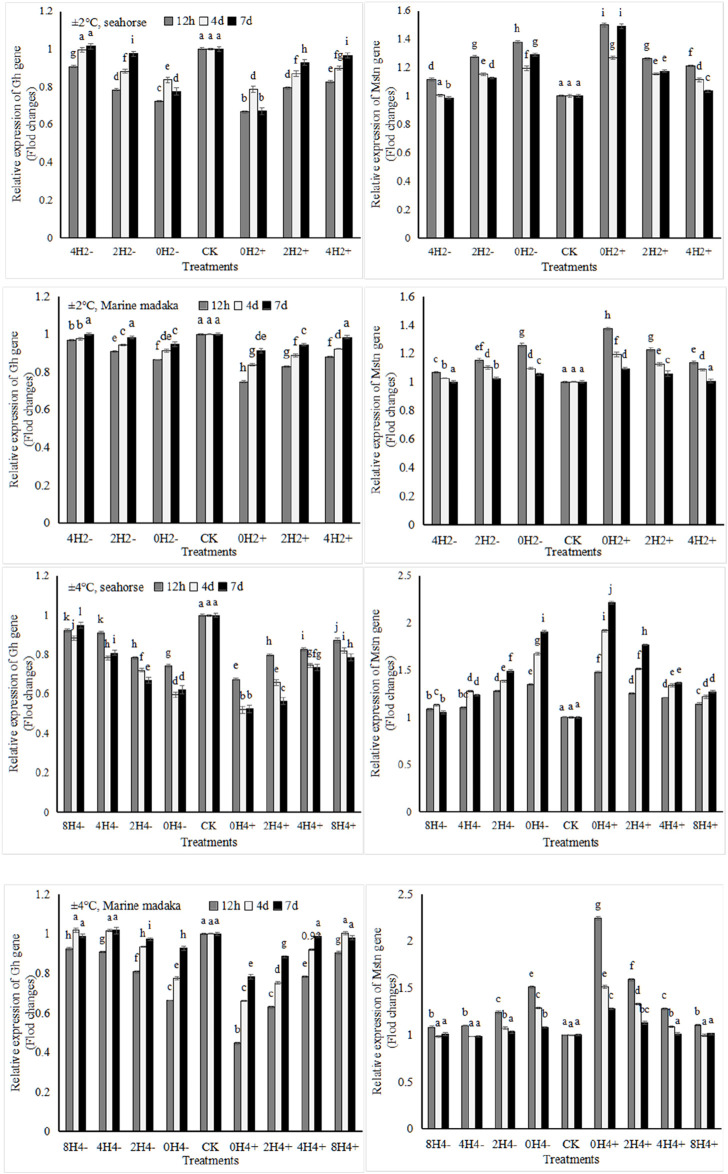
Variations in relative expression levels of *Gh* and *Mstn* genes in *H. kuda* and *O*. *melastigma*.

Two growth-related genes (*Gh* and *Mstn*) were analyzed in the experiment. They were also similar in both fish (Figure 5). Compared to the control treatment, *Gh* was slightly downregulated and *Mstn* was slightly upregulated with temperature changes. Under 2°C amplitude, the expressions of *Gh* and *Mstn* in *O*. *melastigma* were a slight change at the beginning, and had recovered at the end of the experiment. However, their expressions in *H. kuda* were impacted in all treatments at the beginning, and had recovered only in 4H2± on the 7 day. Under 4°C amplitude, their expressions were comprehensively affected in *H. kuda*, while had an obvious impact only in the high-speed treatments of *O*. *melastigma*.

## 4 Discussion

In this study, the results revealed that the growth of farmed fish had an impact by the rapid changes of temperature in the short term. Different fish was affected differently, which reflected in their adaptabilities to changing amplitudes and changing speeds of temperature. The smaller the amplitude and slower the speed was, the less impact it had, making it easier to recover from stress. Compared to the slowly-adapted *H. kuda*, *O*. *melastigma* had a smaller impact and faster recovery. Here, we analyzed the differences between them.

### 4.1 Growth and expressions of growth genes

The growth is the process of synthesizing and accumulating proteins, thereby achieving an increase in indicators such as body weight and body length. The expression of growth-related genes can directly or indirectly reflect protein synthesis in organisms (Luo et al., 2023, p.e81858). MSTN (myostatin) is a muscle growth inhibitor produced by the expression of *Mstn* gene, and is an important regulatory protein that inhibits muscle growth and development (Luo et al., 2023, p.e81858). GH (growth hormone) produced by the expression of *Gh* gene, which can accelerate cell division, and promote protein synthesis (Luo et al., 2023, p.e81858). Both *Mstn* and *Gh* play important regulatory roles in the growth and development of fish (Pan et al., 2022, p.1084). In this study, under the stresses of rapid temperature changes, *Gh* was downregulated and *Mstn* was upregulated in both fish, which indicated the growth of both fish had been affected. There was a good correlation between both genes expressions and the speeds of temperature changes. This indicated that the fish slowed down its cell division and reduced protein synthesis as the changing speed increased (Pan et al., 2022, p.1084), diverting some energy originally used for growth to alleviate the stress, leading to a slowdown in growth (Rasmussen and McLean, 2004, p.397; Pan et al., 2022, p.1084).

Many studies had used the ratio of RNA/DNA as an indicator to evaluate the growth status of fish, such as *Sardina pilchardus* (Ramirez et al., 2004, p.57), *Labeo rohita* (Siddiqua and Khan, 2022, p.e738114), and so on. Generally, RNA is highly expressed in rapidly growing fish, and DNA is relatively stable (Siddiqua and Khan, 2022, p.e738114). A high RNA/DNA ratio indicated there was a fast growth of fish (Siddiqua and Khan, 2022, p.e738114). In addition, the ratio was also highly consistent with the relative expressions of growth-related genes (*Gh* and *Mstn*) (Pan et al., 2020, p.1421). According the ratio and the expressions of both genes, we found the growth of *O*. *melastigma* was rapid recovery in the experiment, but the growth of *H. kuda* was severely inhibited, and the degree was increased with increasing amplitude or speed of temperature changes. The rapid recovery/adaptation ability of *O*. *melastigma* had also been reported in other species, such as *O*. *sinensis*, whose growth was not significant difference under the stress of temperature changes (±8°C, for 15 days) (Fang, 2021). In this study, the growth inhibition of *O*. *melastigma* only appeared in 0H4+ under 4°C amplitude. However, there was a critical line of growth difference observed in *H. kuda*, which was 2°C amplitude +1°C/h speed with 7 day, or 4°C amplitude +1°C/h speed with 4 day. Obviously, *O*. *melastigma* was more adaptable to the stresses caused by rapid temperature changes than *H. kuda*.

In fact, growth was a comprehensive performance that was related to all internal and external conditions, e.g., food. Usually, low food intake inhibited *Gh* and promoted *Mstn* to slow down the growth (Jackson et al., 2020, p.113319). So that, the fish not only required more energy to resist stresses (Portz et al., 2006, p.125), their poor physiological and health state in turn affected their appetite and led to a decrease in food intake, resulting in a decrease in growth rate.

### 4.2 Feeding and nutrition metabolism

Many studies had delved into the impact of temperature on fish’s feeding, indicating that changes in temperature could affect its digestive tract homeostasis, thereby affecting feeding quantity and feeding behavior (Sharma et al., 2017, p.134; Folkedal et al., 2012, p.350). Therefore, the changes of feeding quantity or behavior can be used to evaluate the impact of temperature stresses in fish. For example, temperature stress had a significant impact on the feeding rate and feed conversion rate of *Cyprinus carpio* (Chen et al., 2022, p.737636), *Seriola lalandi* (Stone et al., 2022, p.738567), and *Scophthalmus maximus* (Aydn et al., 2021, p.736891). Our previous researches also observed an increase in feeding response time and a gradual decrease in feeding rate and frequency for *H. kuda* with increasing temperature changes (Pan et al., 2020, p.1084). In this study, both the amplitude and speed of temperature changes had an impact on the feeding behavior of both fish, included prolonged the feeding response time, decreased the feeding rate and the food intake. Actually, the impact was relatively limited on *O*. *melastigma*, while obviously decreased the food intake in almost treatments of *H. kuda* (Table 3). It indicated that rapid temperature changes had a greater impact on the feeding of *H. kuda*.

In fact, a decrease in food intake was an external manifestation of internal physiology, which included the enzyme activities and gene expressions related to nutrition and metabolism. Under rapid temperature changes, the homeostasis in fish was affected to a certain extent. In order to adapt to new environmental conditions, the fish had to expend high energy costs for adaptive regulation (Portz et al., 2006, p.125). Carbohydrate and lipid were the two main energy substrates in fish (Portz et al., 2006, p.125). Therefore, it was of great significance that exploring the impact of environmental changes on the activities of key enzymes mainly involved in carbohydrate and lipid metabolisms and the relative expressions of genes encoded the key enzymes. This study analyzed two key enzyme genes encoding carbohydrate metabolism, *Idh3b* and *Mdh1*, and two key enzyme genes encoding lipid metabolism, *Cpt1* and *Fas*, as well as two endogenous digestive enzymes, AMS and LPS, to compare the effects of temperature stresses on energy metabolism of *O*. *melastigma* and *H. kuda*.

Endogenous digestive enzymes were important indicators for studying the digestion and utilization of nutrients. The enhancement of their activities can promote the absorption and utilization of nutrients in fish (Jiang et al., 2021, p.57). It was generally believed that sugar was short-lived energy substances that underwent rapid decomposition through the action of AMS enzyme. Lipids, on the other hand, were long-lived energy substances that decomposed by LPS enzyme. In this study, the activities of AMS and LPS in different treatments had a significant change. Based on data of food intake, it was speculated that both fish may have increased their digestive enzyme activities, thereby increasing the assimilation efficiency of foodborne sugars and lipids to cope with excessive energy consumption (Tan et al., 2016, p.637). After a prolonged period of 4 or 7 days with severe stress, energy supplementation from carbohydrates became insufficient, and the consumption of lipids increased, showed by an increase in LPS activity (Figure 1). However, the LPS activity in *O*. *melastigma* and *H. kuda* was significantly different, which had been consistently increasing in *H. kuda* and showed up and down oscillations in *O*. *melastigma*. It indicated that *O*. *melastigma* may have already adapted to the temperature stress, with a relatively balanced assimilation alienation effect in the body. However, *H. kuda* was constantly consuming energy to resist the stress, thus fat-decomposition continued to increase. It was speculated that *H. kuda* will require a longer period to adapt to rapid temperature changes under the same stress.

Glucose was the primary energy substrate preferred by organisms (Portz et al., 2006, p.125). Malic acid dehydrogenase (MDH), generated by the expression of *Mdh1* gene, was a key limiting-rate enzyme in glucose metabolism. Fish regulated carbohydrate metabolism by enhancing MDH activity in the liver to reduce the energy burden (Sharma et al., 2017, p.134; Chang et al., 2020, p.110744). Researches had found that the expression of *Mdh1* was also easily affected in temperature-sensitive organisms. For example, *Cyprinus carpio* who was sensitive to low temperature was significantly lower of *Mdh1* expression than that in cold-tolerant individuals (Liu et al., 2018, p.168). In this study, after 12 h stress of rapid temperature changes, the *Mdh1* expression was rapidly upregulated in both fish, indicating that the fish increased their sugar metabolism levels in order to maintain a normal internal physiological state (Sharma et al., 2017, p.134). Clearly, *O*. *melastigma* had a faster response and earlier recovered at the following 4th or 7th day, while its expression was still increasing or oscillating in *H. kuda* at the same time (Figure 3). Similar results had also been reported in other fish (Chang et al., 2020, p.110744). Based on the results, it was speculated that there was some difference in the nutritional metabolism mechanism between *O*. *melastigma* and *H. kuda* in response to rapid temperature changes. Firstly, *H. kuda* faced a larger energy gap under the stress, secondly, aerobic carbohydrate metabolism was probablly the main energy pathway for *H. kuda* to resist stress (Sharma et al., 2017, p.134), so did *O*. *melastigma*, but *O*. *melastigma* was relatively stable and less affected under the same conditions. In a word, *O*. *melastigma* had a stronger adaptability and its more sources of energy than *H. kuda* to deal with rapid temperature changes (Chang et al., 2020, p.110744).

Lipid was also important energy storage substances in fish, and the decomposition of fatty acids can be accompanied by a large amount of energy release. Similarly, the mobilization and decomposition of stored lipids were strictly regulated by various internal and external factors (Cheng et al., 2017, p.110744; Sharma et al., 2017, p.134). The liver also was the main site of lipid metabolism in fish. Before fatty acids entered its decomposition pathway, they needed to be catalyzed by carnitine palmitoyltransferase 1 (CPT1) to form acyl CoA, which then entered the mitochondria β-oxidation reaction. CPT1, generated by the expression of *Cpt1* gene, was the main limiting-rate enzyme in this process (Sharma et al., 2017, p.134). In this study, the expression of *Cpt1* in both fish was affected by temperature changes. *H. kuda* showed an upregulated response in all treatments under 2°C ampitude and in the low-speed treatments under 4°C ampitude, and downregualted response (expression inhibition) in the high-speed treatments. Moreover, *Cpt1* in *O*. *melastigma* showed little or no response under 2°C ampitude, and quickly upregulated and recovered under 4°C ampitude (Figure 4). It can be seen that *Cpt1* gene in *H. kuda* was obviously upregulated in the treatments with slight temperature changes, which indicated that *H. kuda* increased the level of lipid metabolism in the liver, providing more energy for maintaining normal physiological activities (Sharma et al., 2017, p.134). In evidence, it also exceeded its regulatory capacity with large temperature changes. In *O*. *melastigma*, carbohydrate metabolism could ensure energy supply under slight changes. The lipid metabolism was only called on under large changes of temperature. Based on the above analysis, it indicated that the fish provided more additional energy for further adaptation to stress by upregulating the level of fatty acid decomposition (Volkoff et al., 2005, p.3). It also indicated that in the response to rapid temperature changes, the utilization mechanism of fatty acids in both fish was the same, but the utilization efficiency was significant difference.

### 4.3 Antioxidation and immunity

Generally, environmental stresses made the production of intracellular reactive oxygen species (ROS) increasing, and organisms had to consume a large amount of energy from sugar and lipid decomposition to improve their antioxidant defense system. This system included antioxidant enzymes and antioxidant active substances, which were used to clear excess intracellular ROS and maintain normal physiology (Pan et al., 2022, p.1421). As an important member, SOD participated in the catalytic decomposition reaction of ROS, and its activity was an important indicator for evaluating the antioxidant capacity and health state of organisms (Pan et al., 2022, p.1421). MDA was a product of lipid peroxidation in cells and widely used to evaluate the degree of oxidative stress in organisms (Pan et al., 2022, p.1421). In addition, oxidative stress and immunity were closely related, jointly maintaining normal physiology (Fan et al., 2019). In this study, the antioxidant and immune responses of *O*. *melastigma* and *H. kuda* under rapid temperature changes were analysed and compared. Obviously, rapid temperature changes had a significant impact on the SOD activities and MDA contents in the 2 fish. Many similar results had been reported (Tan et al., 2016, p.637; Shi et al., 2022, p.1), indicating that the stress of rapid temperature changes can also induce antioxidant responses in both *O*. *melastigma* and *H. kuda* in this study. However, the timing of inducing antioxidant responses was different. In *H. kuda*, there was a significant increase in SOD activity and MDA content in the treatments with >1°C/h of changing speeds of temperautre, indicating that the stresses in the high-speed treatments may cause more serious oxidative damage to *H. kuda*. For the same reason, the critical safe speed of temperature changes in *O*. *melastigma* was relatively much higher.

Phosphatases, included ACP and AKP, were important metabolic and immune regulatory enzymes in organisms. They can enhance non-specific immune ability in fish (Fan et al., 2019, p.58). Temperature changes can affect their activity fluctuations (Ning et al., 2016, p.117). In this study, AKP activity did not change significantly in the 2 fish under small ampitude. However, it had significantly decreased with increasing changing speeds in *H. kuda* under 4°C ampitude of temperature, making it difficult to recover healthy, indicating that rapid temperature changes possibly inhibited the non-specific immune function of *H. kuda*, also happened in the 0H4± treatments of *O*. *melastigma*. In fact, temperature changes and duration of stress had a significant impact on AKP activity in fish (Meng et al., 2020, p.122). So, the non-specific immune abilities produced by *O*. *melastigma* and *H. kuda* in response to different amplitudes and speeds of temperature changes were different, and their antioxidant abilities were also different, resulting in different outcomes.

The enzymes involved in antioxidation and immunity were synthesized through the expressions of related genes. There were also some differences in the expression of *Sod* gene between *O*. *melastigma* and *H. kuda*. *H. kuda* had a significant expression response to slight temperature changes, while showed an inhibition and downregulation to large temperature changes. On the contrary, *O*. *melastigma* had little response to slight stress, and had strong regulatory ability against large stress. The expression of *Gst* gene, for synthetizing glutathione thiol transferase (GST) (Hooper et al., 2014, p.26), had a similar trend to that of *Sod*. The rapid upregulation of *Gst* was observed in *O*. *melastigma* treatments with 2°C amplitude and in *H. kuda* treatments with 4°C amplitude. All results indicated that *H. kuda* was more sensitive and had limited regulatory ability to rapid temperature changes in this study. This had been confirmed in terms of food supplementation, energy metabolism, and growth performance. In this study, the expression of heat shock proteins (HSPs) and its related genes (*Hsp70*, *Hsp90*) also were analyzed and compared. HSPs can repair cell damage caused by oxidative stress (Iwama et al., 1998, p.35). When the fish felt abnormal stress signals, its heat shock response was activated, and the expression of *Hsp* gene was upregulated, starting to produce HSPs to repair cell damage (Iwama et al., 1998, p.35; Chu et al., 2022, p.528). Therefore, the *Hsp* expression level can reflect the degree of stress response in organisms (Iwama et al., 1998, p.35; Chu et al., 2022, p.528). This mechanism was widely present in aquatic organisms, such as *Chlamys farreri* (Liu et al., 2013b, p.462), *Procambarus clarkii* (Xu et al., 2017, p.9), and *Pampus argenteus* (Shi et al., 2022, p.1). Temperature fluctuations, caused *Hsp* genes expression, and their expression levels were related to the stress-regulation mechanism of organisms under temperature stress (Cui et al., 2013, p.919; Shi et al., 2022, p.1). In this study, the expression level of *hsp70* in *H. kuda* gradually increased with the increase of speed of temperature changes, but without significant changes in *O*. *melastigma*, further indicating that rapid temperature changes caused significant oxidative damage to *H. kuda*.

In this study, the variations in cell apoptosis signaling pathways were also analyzed and compared in *O*. *melastigma* and *H. kuda*. Apoptosis was the main pathway for maintaining the stability of the internal environment in organisms, and was controlled by genes for autonomous and orderly cell death (Zhou et al., 2020, p.735681). It was one of the important forms of non-specific immune system response and regulation to environmental stress (Zhou et al., 2020, p.735681). When a fish was subjected to stress, abnormal stress signals would activate its cell apoptosis signaling factors, inducing the activation of cell apoptosis signaling pathways (Zhou et al., 2020, p.735681). Among them, P53 (tumor suppressor protein) was an important component of the cell cycle signaling process (Siikavuopio and Sather, 2006, p.351). Caspase3 (CASP3), a key enzyme, was widely involved in processes such as the P53 signaling pathway and cell apoptosis pathway (Siikavuopio and Sather, 2006, p.351). In this study, rapid temperature changes had a significant impact on the relative expression levels of *P53* and *Casp3* genes in the liver of *H. kuda*, with significantly increased expression levels and gradually upregulated with increasing amplitude and speed of temperature changes, indicating that the process of apoptosis in liver cells of *H. kuda* had been activated (Liu et al., 2019, p.1407; Zhou et al., 2020, p.735681). The expressions of these 2 genes were also upregulated in *O*. *melastigma*, mainly showed by up and down oscillations and quick recovery. Overall, the expression levels of *P53* and *Casp3* in *H. kuda* were higher than those in *O*. *melastigma*, indicating that the transmission of apoptotic signals in liver cells of *H. kuda* was more responsive to rapid temperature changes than that in *O*. *melastigma*, which was possibly related to the fact that *H. kuda* was more sensitive to temperature changes (Dawood et al., 2022, p.397).

Interleukin-10 (Il-10) was an important inhibitory factor, and played an important role in the inflammation and immune regulation of organisms (Hossain et al., 2022, p.101038). In this study, the *Il-10* expression in *O*. *melastigma* was regular, but chaotic in *H. kuda*. (Pan et al., 2020, p.1421) speculated that the main reason was the disruption of immune system in *H. kuda* caused by rapid temperature changes and its stress. Because of thermal stimulation, the expression of *Il-10* as an anti-inflammatory factor fluctuated rapidly and disorderly, which probably was an expression of transitioning from normal physiological stress into pathological stress in *H. kuda*, represented a weakened response to anti-inflammation and a significant decrease in non-specific immune disease resistance (Liu et al., 2013a, p.462). It was generally believed that the intestine was the central organ of biological stress response. Pathological stress induced pathological changes of intestinal tissues, mainly manifested as mucosal erosion, ulcers, bleeding, and structural damage to mesenteric lymph nodes, leading to a decrease in immune function (Liu et al., 2013b, p.462), and ultimately an increase in disease incidence. This speculation was consistent with the actual situation in production (Wang et al., 2020, p.730). Under rapid temperature changes, *H. kuda* was likely to experience anorexia, then developed into enteritis and death.

## 5 Conclusion

From the results, it can be seen that temperature changes had an impact on both *O. Melastigma* and *H. Kuda*. And *H. kuda* was more sensitive. Under a 2°C amplitude of temperature, *H. kuda,* as a slowly-adapted fish, had significant differences among different speeds of temperature changes, and existd a critical safe speed was about 1°C/h, while the fast-adapted *O. Melastigma* had no effect in all treaments. Under 4°C amplitude, the critical safe speed of *H. kuda* decreased to about 0.5°C/h, while *O. Melastigma* only had a slight impact in the heating treatment with direct input. The reason was comprehensive, including decreased food intake capacity, weakened immune function, increased energy consumption for stress resistance, and so on. The key reason was that *H. kuda* had a single energy supply pathway, which mainly relied on carbohydrate decomposition.

Based on the biological characteristics of *H. kuda,* its precise aquaculture technique needs a stricter and more precise control of environmental temperature to prevent rapid and big temperature changes from affecting the growth and survival.

## Data Availability

The original contributions presented in the study are included in the article/supplementary material, further inquiries can be directed to the corresponding author.
